# Design and Usability Evaluation of Social Mobile Diabetes Management System in the Gulf Region

**DOI:** 10.2196/resprot.4348

**Published:** 2016-09-26

**Authors:** Turki Alanzi, Robert Istepanian, Nada Philip

**Affiliations:** ^1^ Health Information Management and Technology Department College of Applied Medical Sciences University of Dammam Dammam Saudi Arabia; ^2^ Department of Electrical and Electronic Engineering Imperial College London United Kingdom; ^3^ Medical Information and Network Technologies Research Centre Kingston University London United Kingdom

**Keywords:** mobile health, mobile diabetes management, social networking for health care, diabetes mellitus, telemedicine, electronic health, Kingdom of Saudi Arabia

## Abstract

**Background:**

The prevalence of diabetes in the Gulf States is one of the highest globally. It is estimated that 20% of the population in the region has been diagnosed with diabetes and according to the International Diabetes Federation (IDF), five of the IDF’s “top 10” countries for diabetes prevalence in 2011 and projected for 2030 are in this region. In recent years, there have been an increasing number of clinical studies advocating the use of mobile phone technology for diabetes self-management with improved clinical outcomes. However, there are few studies to date addressing the application of mobile diabetes management in the Gulf region, particularly in the Kingdom of Saudi Arabia (KSA), where there is exponential increase in mobile phone usage and access to social networking.

**Objective:**

The objective of this paper is to present the design and development of a new mobile health system for social behavioral change and management tailored for Saudi patients with diabetes called Saudi Arabia Networking for Aiding Diabetes (SANAD). A usability study for the SANAD system is presented to validate the acceptability of using mobile technologies among patients with diabetes in the KSA and the Gulf region.

**Methods:**

The SANAD system was developed using mobile phone technology with diabetes management and social networking modules. For the usability study the Questionnaire for User Interaction Satisfaction was used to evaluate the usability aspect of the SANAD system. A total of 33 users with type 2 diabetes participated in the study.

**Results:**

The key modules of the SANAD system consist of (1) a mobile diabetes management module; (2) a social networking module; and (3) a cognitive behavioral therapy module for behavioral change issues. The preliminary results of the usability study indicated general acceptance of the patients in using the system with higher usability rating in patients with type 2 diabetes.

**Conclusions:**

We found that the acceptability of the system was high among Saudi patients with diabetes, and ongoing work in this research area is underway to conduct a clinical pilot study in the KSA for patients with type 2 diabetes. The wide deployment of such a system is timely and required in the Gulf region due to the wide use of mobile phones and social networking mediums.

## Introduction

The global prevalence of diabetes is alarming, with approximately 366 million individuals living with this long-term condition. The prevalence of diabetes in the Gulf States is one of the highest globally; it is estimated that 20% of the population has been diagnosed with diabetes. According to the International Diabetes Federation (IDF), five of the IDF’s “top 10” countries for diabetes prevalence in 2011 and of those projected for 2030 are in this region [1].There are different causes of such high prevalence, including social norms behavior, climate, diet, and lack of exercise [2].

The Kingdom of Saudi Arabia (KSA) has the seventh-highest prevalence of diabetes in the world; an estimated 20% of the population has been diagnosed with diabetes, most of the type 2 form [1]. However, the recent economic growth in KSA has significantly affected the living standards of the population, leading to adoption of unhealthy eating habits with limited physical activity [2]. Furthermore, type 2 diabetes mellitus prevalence has been increasing as a result of this lifestyle change among the Saudi population [3]. Managing diabetes in the KSA is a challenging task. Various factors contribute to this chronic disease’s prevalence, such as family history, obesity, smoking habits, limited health awareness, social behavioral and culture norms, and health education. As a result, these factors along with coronary artery disease have become a major health burden in the Kingdom. According to the World Health Organization (WHO), it is estimated that noncommunicable diseases will be the principal cause of death in the Kingdom [4].

In recent years, there have been an increasing number of studies on the effectiveness of mobile diabetes management systems globally [5,6]. A recent meta-analysis study indicated the effectiveness of these technologies for both type 1 and type 2 diabetes management and improved glycated hemoglobin (Hb_A1c_), especially for patients with type 2 diabetes [7].

Similarly, social networking has also become an important medium for exchanging health care information between users and patients in recent years [8]. For example, social sites like PatientsLikeMe aim to create a community Web environment for patients, nurses, and society to provide medical information and education, empower patients to share experiences, explore their medical conditions, symptoms, and routines, and support each other [9]. Other social networking health care sites including CureTogether, MedHelp, and mCare provide different health services supported by their delivery models [10-12].

A recent survey of existing social networks for health care illustrates the influence of social networking on health care outcome models [13]. It classified social network services into three categories: (1) health care social networking; (2) consumer personalized medicine; and (3) quantified self-tracking with four major health care interventions offered to the clients through the social networks including clinical trial access, emotional support and information sharing, quantified self-tacking, and questions and answers with a professional physician.

However, most of these studies are conducted for use in the United States, Europe, and developed countries. Although the Gulf region has one of the highest users globally of mobile phones and social networking sites such as Facebook and Twitter [14], to date there is no study on the use of social networking in the region. A recent review [15] by the authors on the use of mobile diabetes management systems and social networking in the Gulf countries found that only three studies have been published to date [16-18]. A study in Bahrain examined the use of mobile phones among a group of patients with diabetes with improved outcomes in Hb_A1c_ [16]. Another study in Iraq examined the feasibility and acceptability of text messages (short message service, SMS) with improved outcomes in diabetic patients’ education [17]. The study in Qatar outlined a system for assisting people with diabetes and managing diabetes through glucose monitoring and diet management that indicated improved satisfaction of diabetic patient users [18]. These few pilot studies indicate that there is an urgent need for further work in the region considering the major challenges of the diabetes epidemic.

Furthermore, no study to date has examined the combination of using social networking and mobile diabetes management tailored specifically for Saudi patients. In this paper, we present the general structure of a mobile diabetes management system tailored for Saudi patients called Saudi Arabia Networking for Aiding Diabetes (SANAD). We also present a usability study of the acceptability of the SANAD system among Saudi patients with diabetes.

## Methods

### System Design

In order to design a mobile diabetes management system for Saudi patients, we conducted a preliminary study on the perception of managing diabetes mellitus through mobile technologies and social networking in the Kingdom [19]. In this study, a mixed-method design with interviews and a survey were used to gather data. Most of the participants were users aged between 10 and 30 years. The outcome of this preliminary study indicated that the acceptance of Saudi patients for using social networking as a tool for better management of their diabetes is relatively high. The acceptance is especially high in the younger population (10 to 30 years) who prefer to use Saudi social networking mediums for managing their condition. Another important finding was that the preferred social networking functionalities such as Ask a Doctor, messaging, blogs, and video tutorials had the highest percentages of suggested functionalities. Furthermore, we found that the proposed management system should include the following key functional components: (1) a mobile diabetes management component; (2) a social networking component; and (3) a behavioral change component.

**Figure 1:**
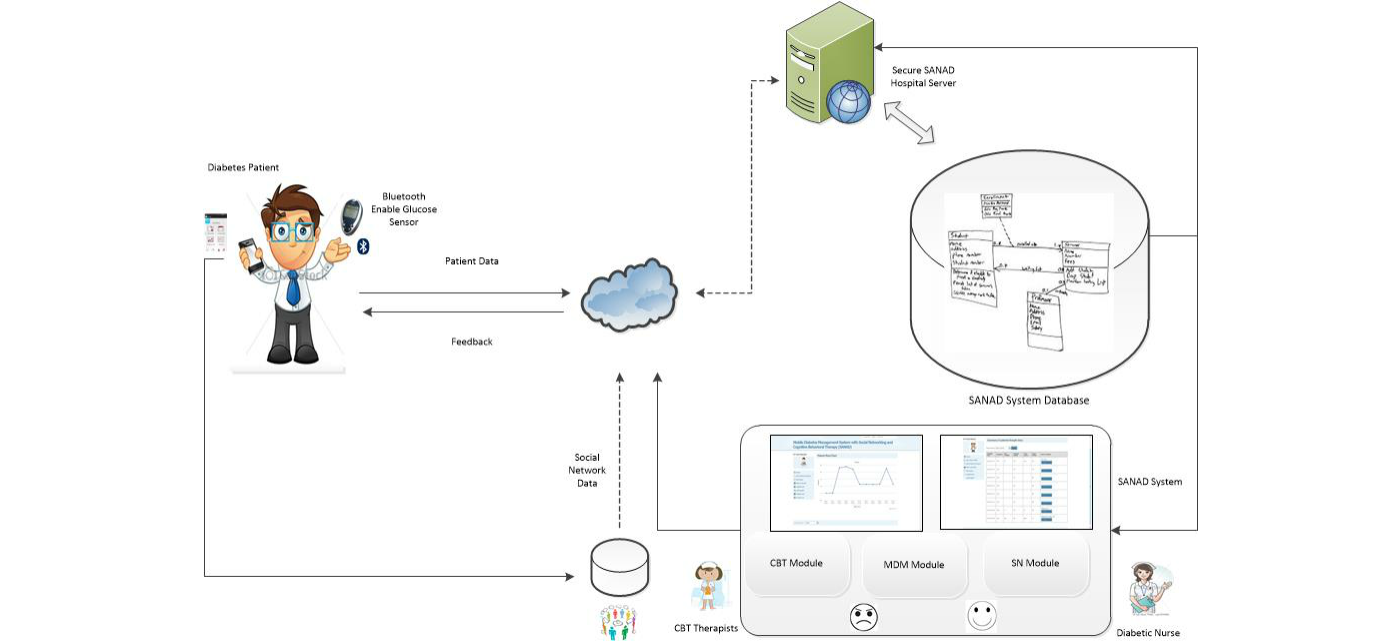
General architecture of the Saudi Arabia Networking for Aiding Diabetes (SANAD) system. CBT: cognitive behavioural therapy; MDM: mobile diabetes management; SN: social networking.

The general architecture of the SANAD system is shown in Figure 1. The architecture consists of three functional components: the mobility module, the social networking module, and the behavioral change function based on cognitive behavioral therapy (CBT). The choice of a CBT module is based on the effectiveness of this approach in diabetes management [20,21].

**Figure 2:**
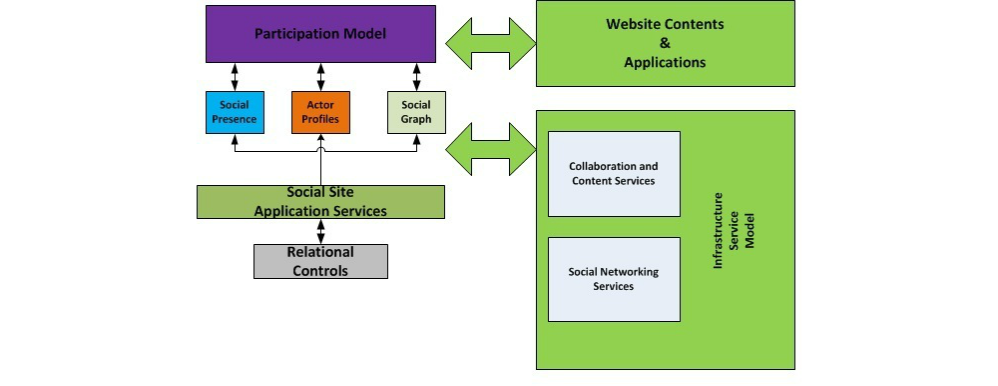
General building blocks of a social networking system.

**Figure 3:**
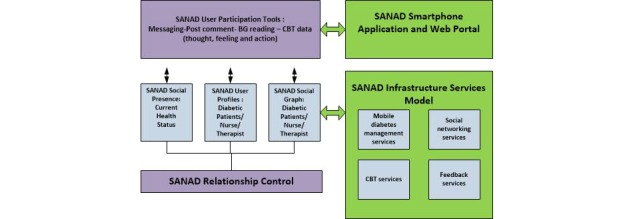
Building blocks of the Saudi Arabia Networking for Aiding Diabetes (SANAD) system. BG: blood glucose; CBT: cognitive behavioral therapy.

The building blocks of a general social networking system are shown in Figure 2. These building blocks are relationship control, social graph, actor profiles, social presence, a participation model, website contents and app, and an infrastructure services model. A detailed description of these blocks is given elsewhere [22]. Based on this architecture, we developed the building blocks of the SANAD system (Figure 3), which are described below.

#### Relationship Control

Relationship controls define the relationship types users can create with each other [12,22]. The relationship control in SANAD is based on the friend relationship among patients with diabetes, nurses that treat patients with diabetes, and the CBT therapist.

#### Social Graph

The social graph theory used in the SANAD system signifies the following relationships: (1) patients with diabetes with patients with diabetes, (2) patients with diabetes with nurses that treat patients with diabetes, and (3) patients with diabetes with the CBT therapist.

#### User Profiles

User profiles are well known as actor profiles in the general social network. The actor profiles in SANAD are of three types: patients with diabetes, nurses that treat patients with diabetes, and the CBT therapist.

#### Social Presence

Social presence is a new model in social network frameworks. In earlier social networking, social presence was produced by being connected and available. However, nowadays it is well known as a user’s current status, which is a description of a user’s activity.

#### User Participation Tools

Participation tools provide techniques for users to communicate, interact, and participate with other users through instant messaging and message boards. The participation tools of SANAD are messaging between the users, the ability of users to post comments, the ability of patients to insert their reading information, and the ability of users to submit their therapeutic data.

#### Mobile App and Web Portal

A custom server hypertext preprocessor app is used to support remote log-in. It is also used to review patient data and user settings, and to provide such feedback by the medical staff. In addition to viewing patient data and assessment results, a key feature is allowing patients with diabetes access via the Web rather than mobile use. Patient data was stored on a remote Microsoft SQL secure database server portal. The mobile phone platform is implemented using Android operating system (Google, Mountain View, CA, USA) and the Java Software development kit (Oracle Corporation, USA). The blood glucose sensor (LifeScan, Inc ,OneTouch, USA) using Bluetooth (Polymap Wireless adaptor, Tucson, USA) is also used for transferring the data to the mobile app. GALAXY S III (Samsung, South Korea) is used as the mobile phone, running Android 4.0 to send the clinical data in a real-time to the server portal.

#### Infrastructure Services Model

In this model, the content and services of the SANAD system are presented as mobile diabetes management, social networking, CBT, and feedback services.

### Usability Study

A preliminary evaluation study of the SANAD system was carried out in the Dammam region in the KSA in collaboration with the medical school in the region. The main objective of the study was to investigate the usability aspects of the SANAD system among the Saudi patients with diabetes. A total of 33 patients with type 2 diabetes (17 male, 16 female) participated in the study. Patients were recruited by clinical staff during an office visit or by sending a text message.

Three tasks were designed based on the functional components carried out by the SANAD system. Care was taken to ensure that the tasks were simple and met the purpose of the app. The tasks are described in Textbox 1.

**Tasks designed based on the functional components of the system.**

Task

Perception toward the SANAD mobile diabetes management module

- measuring blood glucose level by using the module
- sending it to the the Saudi Arabia Networking for Aiding Diabetes (SANAD) mobile server

Perception toward the SANAD social networking module services

- sending a private message to the nurse or other friend
- watching videos
- searching
- finding a friend

Perception toward the SANAD cognitive behavioral therapy (CBT) module

- submitting their CBT data to the server
- the system displaying the data in chart and tables

The Questionnaire for User Interaction Satisfaction (QUIS) was used for designing the survey questionnaires. QUIS was developed by Shneiderman and is based on an Object-Action Interface (OAI) model [23]. The assessment of the satisfaction of the users is subjective and complex, so QUIS was used because it gauges the users’ satisfaction with the software’s usability in a standard, reliable, and valid way. QUIS was initially implemented using a 9-point Likert scale rating in a standard paper-and-pencil form. It focuses on the analysis of usability based on overall reaction to the system, screen factors, terminology, system feedback, learning factors, and system capabilities [24]. The QUIS version 7.0 was used and the questionnaire was arranged in a hierarchical format which included a demographic questionnaire and six scales for measuring overall reaction ratings of the system. Each item in the QUIS questionnaire was rated on a scale of 1 to 9, and an additional option of not applicable (N/A) was also provided [25]. The general characteristics of the participants are presented in Table 1.

**Table 1:** Patient demographics of the usability study (N=33).

General characteristics			Type 2 diabetes, n (%)
Gender			
	Male	17 (48)	
	Female	16 (52)	
Age group, years			
	18-40	14 (42)	
	41-50	18 (45)	
	51-65	1 (3)	
Level of education			
	Secondary	16 (48)	
	Diploma	7 (21)	
	University or more	10 (30)	
Marital status			
	Married	12 (36)	
	Widowed/divorced	12 (36)	
	Never married	9 (27)	
Diagnosed with diabetes			
	≤5 years	13 (39)	
	6-10 years	8 (24)	
	11-15 years	5 (15)	
	>15 years	7 (21)	

## Results

**Figure 4:**
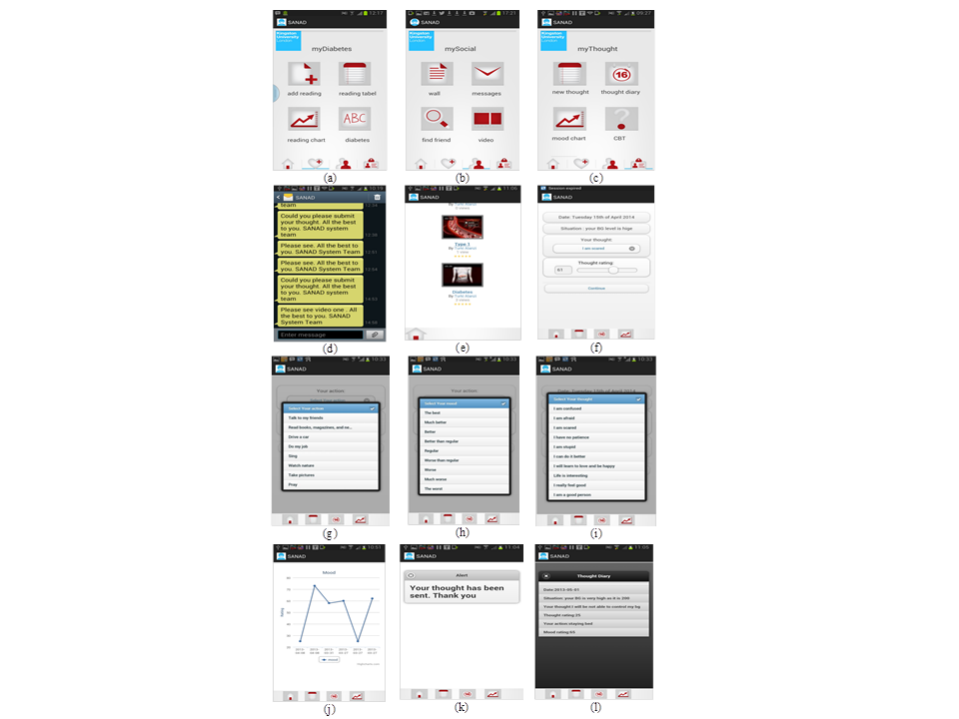
Snapshots of the diabetic patient mobile phone interfaces in the Saudi Arabia Networking for Aiding Diabetes (SANAD) system.

**Figure 5:**
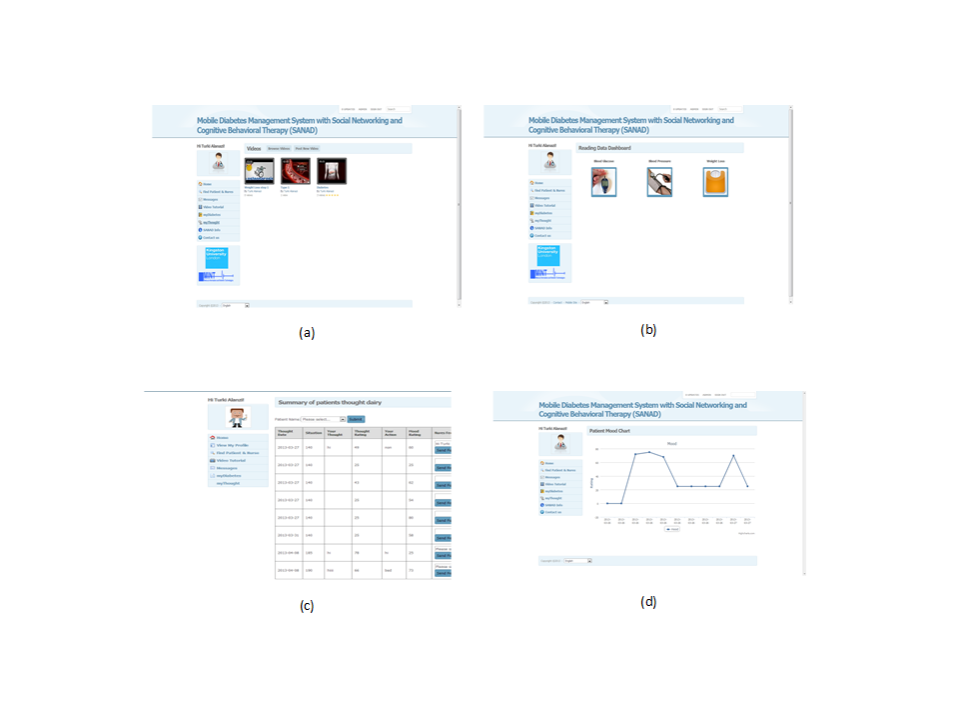
Snapshots of the diabetes nurse portal interface end (top panel) and snapshots of cognitive behavioral therapist’s portal interface end (bottom panel) in the Saudi Arabia Networking for Aiding Diabetes (SANAD) system.

The SANAD system consists of two main components. First, the patient end is comprised of the SANAD mobile app. The second is a remote Web portal hosted in a hospital. The mobile app facilitates sending, receiving, and reviewing patient’s diabetic data whereas the Web portal app provides framework to the diabetic and the CBT specialists to set the reading schedule, review the patient’s statue performance and adherence, and provide a suitable feedback to the patient using text messages. Samples of the app’s interfaces of the SANAD system are shown in Figure 4 and samples of the nurse and CBT therapist’s portal interfaces are shown in Figure 5. The concept of the SANAD system is based on four modules: the mobile diabetes management module, the social networking module, the CBT module, and the feedback mechanism and messaging box module.

### Mobile Diabetes Management Module

The key function of the mobile diabetes management module is to provide the mobility component of the SANAD building blocks. This module consists of patient’s mobile diabetes component, which assists the patient to send their blood glucose data remotely via their mobile phone and display their blood glucose data graphically (eg, tables and charts). In addition, it consists of a Web portal medical staff end, which assists in scheduling the reading time and date, sending feedback, and observing patient status remotely.

### Social Networking Module

The key function of the social networking module is to provide the necessary social information required for the SANAD system. This includes sharing information and providing emotional support among the users. This module consists of a patient’s mobile social networking end, which provides a simple mechanism for interactivity between the patients and the clinicians, displays video education tutorials on diabetes, and contains a Web portal medical staff end, which assists them to interact with patients and post video education tutorials and useful information.

### The CBT Module

The key function of this module is to provide the behavioral change component of the SANAD building blocks. This module consists of a patient’s mobile CBT end, which assists the patient to send their CBT data remotely via the mobile phone and to display their CBT (thoughts, feelings, and actions) in graphic presentations (eg, tables and charts). In addition, it consists of a Web portal CBT therapist’s end, which assists in sending feedback and observes the status of the patient’s behavioral change remotely. This module aids in CBT intervention by applying a classification algorithm to decide whether to trigger an intervention text message telling the patient to submit CBT data.

### Feedback Mechanism and Messaging Box Module

The key function of this module is to provide the necessary gate for the medical staff and the CBT therapist to set and send automated and manual feedback to the patients.

The statistical results of the patient’s perceptions of the SANAD system are shown in Table 2. Preliminary results of this study indicate the general acceptance by patients with type 2 diabetes in using this system. Out of the six items, four were rated lower than the mean response (mean 6.40). These items were ease of use, perceived powerfulness, stimulating, and flexibility. The other two items were rated higher than the mean response. Depending on these results, it can be concluded that the system has a positive impact on patients with diabetes, but the overall impression and the satisfaction in using the system received a good response.

**Table 2:** Overall responses of patients with type 2 diabetes to the six items.

Overall reaction	Mean (SD)
Terrible/wonderful	6.79 (1.24)
Difficult/easy	6.33 (0.92)
Frustrating/satisfying	6.64 (1.32)
Inadequate power/adequate power	6.24 (1.30)
Dull/stimulating	6.39 (1.14)
Rigid/flexible	6.00 (0.97)
Mean	6.40 (N/A)^a^

^a^N/A: not applicable.

## Discussion

### Principal Findings

Results of this study, the first design and usability evaluation study of a social mobile type 2 diabetes management system in Saudi Arabia, provides evidence that SANAD has a potential positive impact to support the management of individuals living with type 2 diabetes.

We expect the addition of a CBT and social networking module within the diabetes management system will be effective in streamlining the lifestyles of the patients accordingly so that the chances or the risks of having diabetes-associated diseases, such as cardiovascular diseases, can be reduced with improved blood glucose monitoring and maintenance. The social networking module improves the user experience in using the mobile app and can be a source for knowledge sharing and query resolving. Integrating these two systems with the diabetes management module (DMS) results in a study with unique characteristics that could help find novel ways of diabetes management. Experienced medical staff including nurses that treat diabetes, a behavioral therapist, medical practitioners, and dietitians, along with high technology infrastructure that supports effective module integration, are used in the study. The overall reaction of the participants to using the system was good, supporting the use of behavioral therapy and social networking in diabetes management. There are studies that focus on using CBT as an intervention in managing diabetes and other diseases [26-28], but to the best of our knowledge, there is no study that integrates CBT and social networking for diabetes management in the region. As there are no prior studies in the region which used CBT and social networking modules for diabetes management, this study can be a major reference in analyzing the usability of the SANAD system. Though the setting is well-built, the results of the study cannot be generalized to all patients with diabetes.

### Limitations

The study is designed and tailored according to the needs and expectations of Saudi patients with diabetes. Therefore the results of the study can be attributed in particular to KSA. As a result, the study may not be applicable in other regions internationally. However, the results can be a source to implement similar studies in the regions which are similar to the Kingdom and where the lifestyles, needs, and expectations of the patients match. Another limitation of the study is that only patients with type 2 diabetes were included. As a result, the study can only be streamlined to patients with type 2 diabetes from the Kingdom and cannot be generalized to all diabetes patients.

The impact of the system will be assessed in a follow-up study. If this study appears to have positive effects, the behavioral intervention along with social networking could be implemented in other care settings in similar regions, for effective mobile diabetes management.

### Conclusion and Future Work

Here, we present a new architecture of CBT for a social mobile diabetes management system (SANAD) tailored for the Gulf region and the KSA. This system consists of three modules: (1) a mobile diabetes management module, (2) a social networking module, and (3) a CBT module. In addition, the method used in the usability evaluation of the study of a mobile app was discussed. The outcome of the usability study indicated that the acceptability of the system was high among Saudi patients with diabetes. In particular, patients with type 2 diabetes reported higher satisfaction with the overall impression, satisfaction, being stimulated, ease of use, perceived powerfulness, and flexibility. Ongoing work in this research area is underway to conduct a clinical pilot study in the Kingdom for patients with type 2 diabetes. The wide deployment of such a system is timely and required in the Gulf region due to the wide use of mobile phones and social networking mediums.

## Abbreviations

CBT: cognitive behavior therapy
Hb _A1c_: glycated hemoglobin
IDF: International Diabetes Federation
KSA: Kingdom of Saudi Arabia
QUIS: Questionnaire for User Interaction Satisfaction
SANAD: Saudi Arabia Networking for Aiding Diabetes
SMS: short message service
